# Course of well-being and mental health in Switzerland during the COVID-19 pandemic: results of a national survey within the framework of the COH-FIT study

**DOI:** 10.3389/fpsyt.2025.1642325

**Published:** 2026-02-16

**Authors:** Christian G. Huber, Janina Billian, Tiziana Ziltener, Philippe Conus, Roland von Känel, Gregor Hasler, Jihed Sehli, Anna Mosina, Damir Krinitski, Erich Seifritz, Eva Kowalinski, Isabelle Gothuey, Jennifer Marian, Undine E. Lang, Trevor Thompson, Marco Solmi, Christoph U. Correll, Sabina C. Heuss, Andres R. Schneeberger

**Affiliations:** 1 Universitäre Psychiatrische Kliniken (UPK) Basel, Klinik für Erwachsene, University of Basel, Basel, Switzerland; 2 Faculty of Psychology, Division of Clinical Psychology and Epidemiology, University of Basel, Basel, Switzerland; 3 Department of Psychiatry, University of Lausanne, Lausanne, Switzerland; 4 Department of Consultation-Liaison Psychiatry and Psychosomatic Medicine, University Hospital Zurich, University of Zurich, Zurich, Switzerland; 5 University of Fribourg, Fribourg Network of Mental Health (RFSM), Fribourg, Switzerland; 6 Clienia AG, Wetzikon Psychiatric Centre, Wetzikon, Switzerland; 7 Clinic for Child and Adolescent Psychiatry and Psychotherapy, University Hospital of Psychiatry Zurich, Zurich, Switzerland; 8 Department of Psychiatry, Psychotherapy and Psychosomatics, Psychiatric Hospital, University of Zurich, Zurich, Switzerland; 9 Réseau Fribourgeois de Santé Mentale, Villars-sur-Glâne, Switzerland; 10 Medicine Section, University of Fribourg, Fribourg, Switzerland; 11 Private Practice, La-Tour-de-Peilz, Switzerland; 12 Centre for Chronic Illness and Ageing, University of Greenwich, London, United Kingdom; 13 Department of Psychiatry, University of Ottawa, Ottawa, ON, Canada; 14 Department of Mental Health, The Ottawa Hospital, Ottawa, ON, Canada; 15 Ottawa Hospital Research Institute (OHRI) Clinical Epidemiology Program, Ottawa, ON, Canada; 16 Department of Child and Adolescent Psychiatry, Charité Universitätsmedizin Berlin, Berlin, Germany; 17 The Zucker Hillside Hospital, Northwell Health, NY, United States; 18 Donald and Barbara Zucker School of Medicine at Hofstra/Northwell, Hempstead, NY, United States; 19 The Feinstein Institute for Medical Research, Center for Psychiatric Neuroscience, Manhasset, NY, United States; 20 University of Applied Sciences and Arts Northwestern Switzerland Fachhochschule Nordwestschweiz (FHNW), School of Business, Olten, Switzerland; 21 University of California San Diego, La Jolla, CA, United States

**Keywords:** COVID-19, mental health, well-being, Switzerland, pandemic (COVID-19), COH-FIT, survey

## Abstract

**Introduction:**

The COVID-19 pandemic significantly impacted the mental health of the Swiss population.

**Methods:**

This study analyzed data from the Collaborative Outcome study on Health and Functioning during Infection Times (COH-FIT) across three pandemic waves: T1 (April–June 2020), T2 (July–December 2020) or T3 (January–June 2021). Each participant participated only once, during one of these three waves. Participants reported their subjective well-being and mental health status for the two weeks prior to the pandemic (pre-pandemic baseline) and during their respective pandemic wave. Subjective well-being was assessed using the World Health Organization Well-Being Index (WHO-5) from 4,037 participants, while mental health was measured via the P-score, completed by 3,375 participants. The WHO-5 ranges from 0 to 100, with higher scores indicating better well-being, while the P-score also ranges from 0 to 100 whereas higher scores represent greater levels of perceived burden across five domains of mental health. Pre- and intra-pandemic differences were analyzed using Wilcoxon tests and ANOVA, with subgroup analyses across seven Swiss regions utilizing the Kruskal-Wallis test.

**Results:**

Participants had a mean age of 45.6 years (61.9% female). Results showed a substantial decline in well-being during the pandemic, with average WHO-5 scores decreasing from 75.3 pre-pandemic, to 66.5 during the first wave, 69.1 during the second, and 65.1 during the third, representing relative reductions of 11.7%, 8.2%, and 13.5%. The percentage of participants at risk for depression (WHO-5 <50) peaked during the third wave at 19.8%, up from 10.0% pre-pandemic. Mental health burden, as measured by the P-score, increased significantly during the first wave (from 20.6 to 27.3, +32.5%), and remained elevated across the two subsequent waves, with no significant recovery observed. Wilcoxon tests indicated significant differences between pre-pandemic and intra-pandemic WHO-5 and P-scores, with the largest effect sizes during the third wave (*r* = 0.652 for WHO-5; *r* = 0.487 for P-score). ANOVA showed significant intra-pandemic differences in WHO-5 across waves (*p* < 0.001), with improvements noted in the second wave. However, no intra-pandemic differences in P-scores were found (*p* = 0.298). Regional analyses revealed that Ticino, the Lake Geneva region, and Northwestern Switzerland experienced the most pronounced declines in well-being and increases in mental health burden. In contrast, Espace Mittelland and Eastern Switzerland experienced a less severe impact.

**Discussion:**

Overall, these findings highlight the considerable and lasting impact of COVID-19 on mental health in Switzerland, emphasizing the need for targeted interventions, particularly in the most affected regions.

## Introduction

The well-being and mental health of the general population in Switzerland deteriorated during the COVID-19 pandemic (1–7). A variety of factors contributed to mental health challenges for populations worldwide throughout the pandemic, such as anxiety about contracting the virus, concerns for loved ones, feelings of uncertainty about the present and future, disruptions of daily routines, restrictions on social interactions, and increased social isolation (8–14). Additionally, economic instability, job losses or changes in working conditions, limited access to mental health services, and constant exposure to distressing news and misinformation were also contributors to mental health problems during COVID-19 (9, 13, 15–24).

Concerning excess deaths (25), Switzerland experienced a low impact of the COVID-19 pandemic in comparison to other countries worldwide (26, 27). Prior to the COVID-19 pandemic, at the beginning of March 2020, excess mortality in Switzerland was recorded at −8% in percentage terms. However, by April 2020, it had reached a preliminary peak of 3%. From April to October 2020, excess mortality declined to approximately 1%. The strongest increase occurred starting in November 2020. From that point until the spring of 2021, excess mortality escalated to 13%. Subsequently, it gradually decreased to 8% beginning in February 2021, where it stabilized.

The incidence of infections and mortality associated with COVID-19 experienced a notable increase beginning in mid-March 2020 (28). This trend reached a temporary plateau in May 2020, at which point nearly 30,000 infections and approximately 1,600 deaths had been reported. Subsequently, in early November 2020, there was another surge in mortality, with deaths rising from approximately 2,000 to 9,200. From February 2021 onward, the increase in fatalities was modest, ultimately resulting in a total of 10,383 deaths by June 30, 2021. During the same timeframe, 700,110 infections were documented.

Globally, the surges of the COVID-19 virus prompted significant restrictions on public life and social interactions to curb its spread. While these measures served to protect public health, they also had detrimental effects on individuals’ mental well-being worldwide. Research has consistently documented a link between the intensity of COVID-19 restrictions and the decline in mental health across diverse populations. In Switzerland, the online survey by de Quervain et al. (2) offers key insights into the psychological effects of Switzerland’s first COVID-19 wave. Conducted between April and June 2020, the study found that nearly half experienced heightened stress, and many reported increased depressive symptoms. Interestingly, a quarter noted decreased stress levels, and anxiety decreased over time. Similarly, Diaz Hernandez et al. (4) examined the pandemic’s initial impact on Switzerland’s population between March and April 2020, showing that one-third experienced worsened mental well-being, and nearly half expressed specific mental health concerns. Schrempft and colleagues (6) found that the peak prevalence of probable depression and anxiety among adults aged 35 and older in Switzerland occurred in February 2021, with a decrease in these symptoms by June 2021 as COVID-19 measures relaxed. By that time, mental health had improved and approached pre-pandemic levels, although many reported a heightened need for psychological support (6). In contrast, Foster et al. (29) found that mental distress increased among young adults during 2021, the second year of the pandemic, leading to chronic perceived stress. This finding suggests that there may be differences among population groups in their responses to the stressors associated with COVID-19, while this study examined the Swiss population as a whole. In summary, these studies showed that mental health in Switzerland declined considerably after the beginning of the COVID-19 pandemic, though there was some variation throughout the pandemic, likely linked to improvements or deteriorations in the pandemic situation and the severity of the restrictions.

Using data from 15 countries worldwide, a study by Aknin et al. (30) demonstrated that stricter policies were associated with heightened psychological distress and diminished life satisfaction, with substantial coefficients indicating a strong relationship. Similarly, an analysis of crisis hotline data in Austria and Germany during the early months of the pandemic revealed a pronounced increase in calls that coincided with the implementation of restrictive measures (31). A subsequent decrease in hotline calls was observed following the lifting of some COVID-19 restrictions, suggesting a direct impact on public mental health (31). Similar trends were observed in Australia, where more stringent restrictions in Victoria led to nearly double the prevalence of clinically significant symptoms of depression and anxiety compared to less restricted regions (32). Collectively, these findings underscore the psychological burden imposed by government interventions during the pandemic.

In Switzerland, the nationwide lockdown was initiated by the Swiss Federal Council in March 2020. From March 2020 to June 2021, various measures were implemented, including restrictions on gatherings, the closure of non-essential businesses, school closures, mask mandates, social distancing guidelines, and recommendations for remote work. These measures were adapted according to the severity of the situation. The first easing of COVID-19 restrictions occurred on April 16, 2020, which included relaxed rules for public transportation.

Switzerland is composed of 26 cantons, each of which was permitted to implement regional measures to manage the pandemic following the nationwide lockdown. Beginning June 19, 2020, cantonal authorities assumed greater responsibility for decision-making. As the epidemic situation deteriorated in the autumn and winter of 2020/2021, cantons enacted varying levels of restrictions, such as regulations on mask-wearing and limitations for restaurants and bars, resulting in a fragmented approach to public health measures within close geographic proximity. A second wave of more stringent nationwide restrictions was enacted by the Swiss Federal Council from January to June 2021, encompassing restaurant closures and prohibitions on gatherings of more than five individuals.

In comparison to other countries, Switzerland had relatively liberal measures in place to combat the spread of COVID-19, as shown by the Oxford COVID-19 Government Response Tracker (33). The tracker employs a stringency index, which is a composite measure adjusted to a scale from 0 (indicating no restrictions) to 100 (representing the strictest measures). The strictest measures were implemented at the onset of the pandemic on March 16, 2020, including school closures and a ban on gatherings of more than five individuals, as mandated by the Bundesrat. Throughout the pandemic, Switzerland’s stringency index fluctuated between a maximum of 73.2 (during the most restrictive period from March 16 to April 26, 2020) and a minimum of 29.2 (the least restrictive level recorded on October 18, 2020). Distinctions between vaccinated and unvaccinated individuals were not made until June 26, 2021. By the end of the third wave on June 30, 2021, the stringency index for vaccinated individuals was recorded at 33.3, compared to 44.4 for unvaccinated individuals. For a graphic illustration of the excess mortality, cumulative confirmed COVID-19 cases and deaths and the COVID-19 stringency index, see the **Supplementary Material**.

Swiss cantons experienced varying impacts from COVID-19, particularly concerning rates of infection, mortality, and the implementation of restrictive measures. The canton of Ticino, predominantly Italian-speaking, emerged as one of the most severely affected regions in Europe, experiencing significant excess mortality varying from varying from 15% to 21% (34). Investigating the mental health repercussions of the pandemic in Ticino, Piumatti et al. (57) reported marked increases in severe depression, anxiety, and stress among the canton’s population from spring 2020 to summer 2021.

Other highly impacted regions in Switzerland included the French-speaking Lake Geneva region (34–36). Consequently, cantonal authorities adopted varied strategies in response to the crisis, with Ticino, Geneva, and Vaud instituting more stringent measures compared to other Swiss regions (35).

Overall, despite Switzerland adopting relatively lenient restriction policies to combat COVID-19 in comparison to other countries worldwide, it is likely they had a negative impact on the quality of life, well-being, and mental health of individuals (1, 3–7, 31, 32, 37, 38).

### Aims of the study and hypotheses

This study employs the COH-FIT cross-sectional anonymous survey, which collected data at the population level, to explore changes in well-being and mental health burdens at three distinct time points during the COVID-19 pandemic in Switzerland in 2020 and 2021, and comparing these outcomes to pre-pandemic levels based on participants’ self-ratings. It represents the first use of COH-FIT data in Switzerland.

Firstly, we expect participants to report worse well-being and mental health during all three pandemic time waves compared to the two weeks prior to the pandemic.

Secondly, while all three intra-pandemic time points are anticipated to show worse well-being and mental health than the pre-pandemic baseline, we hypothesize that well-being will be lowest and mental health burden highest during the initial phase of the pandemic and in early 2021, periods characterized by increased infections and stricter regulations. In contrast, we anticipate that participants will report better well-being and mental health in summer 2020, which corresponds with a relaxation of infection rates.

Thirdly, we assume regional differences in well-being and mental health across Switzerland, anticipating that the inhabitants of Ticino and Lake Geneva region will report worse outcomes.

## Methods

### Survey

The study analyzes the Swiss data from the COH-FIT study (39–43), namely 5,433 adult participants in Switzerland during the COVID-19 pandemic (40–42).

Participants were recruited through convenience sampling, utilizing media releases, and social media platforms, as well as word-of-mouth. Recruitment efforts were supported by the official website of the COH-FIT study, which was launched on April 25, 2020, in English, German, and French, with Italian language added on April 28, 2020, and Romansh language added on May 14, 2020. A press release in four languages was issued on May 14, 2020, with support from Centre Hospitalier Universitaire Vaudois (CHUV), Fribourg University, University of Basel, Fachhochschule Nordwestschweiz (FHNW), and University Hospital Zurich. The survey was conducted between April 2020 and June 2021, with three waves of recruitment: Directly after lockdown, from April to June 2020 (T1), six months later from July 2020 to December 2020 (T2), and the following six months from January 2021 to June 2021 (T3). The survey also included a retrospective assessment of two weeks prior to the onset of the pandemic, approximately from late February to mid-March 2020.

### Measurements

#### WHO-5

Subjective well-being was assessed using the 5-item World Health Organization Well-Being Index (WHO-5; 46). This scale measures well-being in terms of cheerfulness, calmness, activity, feeling rested after waking up, and interest in life (44, 45). Participants were asked to evaluate their well-being over the past two weeks during the COVID-19 pandemic and retrospectively reflect on the two pre-pandemic weeks. Each item was rated on a visual analog scale (VAS) scale from 0 to 100, with raw scores calculated by summing the values of the five items. The raw scores were then divided by five, resulting in a final score ranging from 0 to 100, with higher scores indicating better well-being.

In their systematic review on the WHO-5, Topp et al. (45) found several studies using a cutoff score of ≤ 50 on the WHO-5 to establish a ‘screening diagnosis’ of depression. Other studies applied a stricter cutoff of ≤ 28, reflecting the well-being level of patients with DSM-IV major depression (46, 47). Aggregating findings from all WHO-5 studies on depression screening, the overall weighted sensitivity was 0.86 and specificity was 0.81, both considered acceptable. Therefore, in our sample, a WHO-5 score below the cutoff of <50 indicated a screening for depression and WHO-5 values below <29 indicated potential major depression (40, 41).

#### P-score

Mental health was evaluated using the P-score, a composite index comprised of clinically validated measures based on DSM-5 criteria (48). The P-score encompasses five domains of mental health: anxiety, depression, post-traumatic stress disorder (PTSD), psychosis (including hallucinations and delusions), and psychophysiological factors (covering sleep, focus, and stress).

The P-score was derived from *a priori* identified items that demonstrated validity through external validation against comprehensive questionnaires, achieving a correlation threshold of *r* ≥ 0.5, resulting in a total of thirteen items (40, 41). To compute the P-score, the mean item score for each of the five domains was calculated and then averaged to derive an overall score from 0 to 100, with higher scores indicating a worse mental health status. Participants were also asked to retrospectively report their mental health burden experienced within the two weeks prior to the pandemic to facilitate comparison of the intra-pandemic P-score with the mental health – similar to the approach used for the WHO-5.

### Procedure

Participants completed the WHO-5 and P-score assessments as part of the COH-FIT online survey at one of the three time waves.

Data were analyzed using IBM SPSS Statistics 27.0 (49). The WHO-5 and P-scores were initially described using means and standard deviations for the three distinct intra-pandemic waves, as well as across all participants for the pre-pandemic waves. Percentage changes for both measurements were also calculated. Pre-pandemic WHO-5 and P-scores which participants were asked to evaluate retrospectively, have been aggregated for all participants across the three waves, as they referred to the same two weeks preceding the pandemic.

The significant Kolmogorov-Smirnov (*p* < 0.001) test indicated that neither the WHO-5 nor the P-score were normally distributed. Consequently, we employed non-parametric tests in the subsequent analyses.

We used Wilcoxon signed-rank tests for paired samples to assess differences in WHO-5 and P-scores between the pre-pandemic and intra-pandemic periods among participants who provided data at both time points during the first and second waves. This approach allowed for direct comparison of scores within the same individuals.

We employed Wilcoxon tests for paired samples to evaluate differences between pre-pandemic and intra-pandemic WHO-5 and P-scores for participants during the first and second waves. The analysis included participants who provided data for both pre- and intra-pandemic period, enabling direct comparison.

We conducted two multivariate analyses of variance (ANOVAs) to investigate the effects of time periods on the two dependent variables: WHO-5 and P-score. The three intra-pandemic time periods were treated as fixed factors. Prior to conducting the ANOVA, the data were screened for assumptions, including multivariate normality, homogeneity of variance-covariance matrices, and linearity. The ANOVA employed a significance level of *α* = .05, and the Sidak *post hoc* analysis were used to further explore any significant effects observed in the overall model. Both dependent variables were not normally distributed according to the Shapiro-Wilk test; the P-score was positively skewed, while the WHO-5 was negatively skewed. Therefore, in addition to the two ANOVAs, a Kruskal-Wallis test was conducted, which confirmed the results of the ANOVAs.

**Figure 1:**
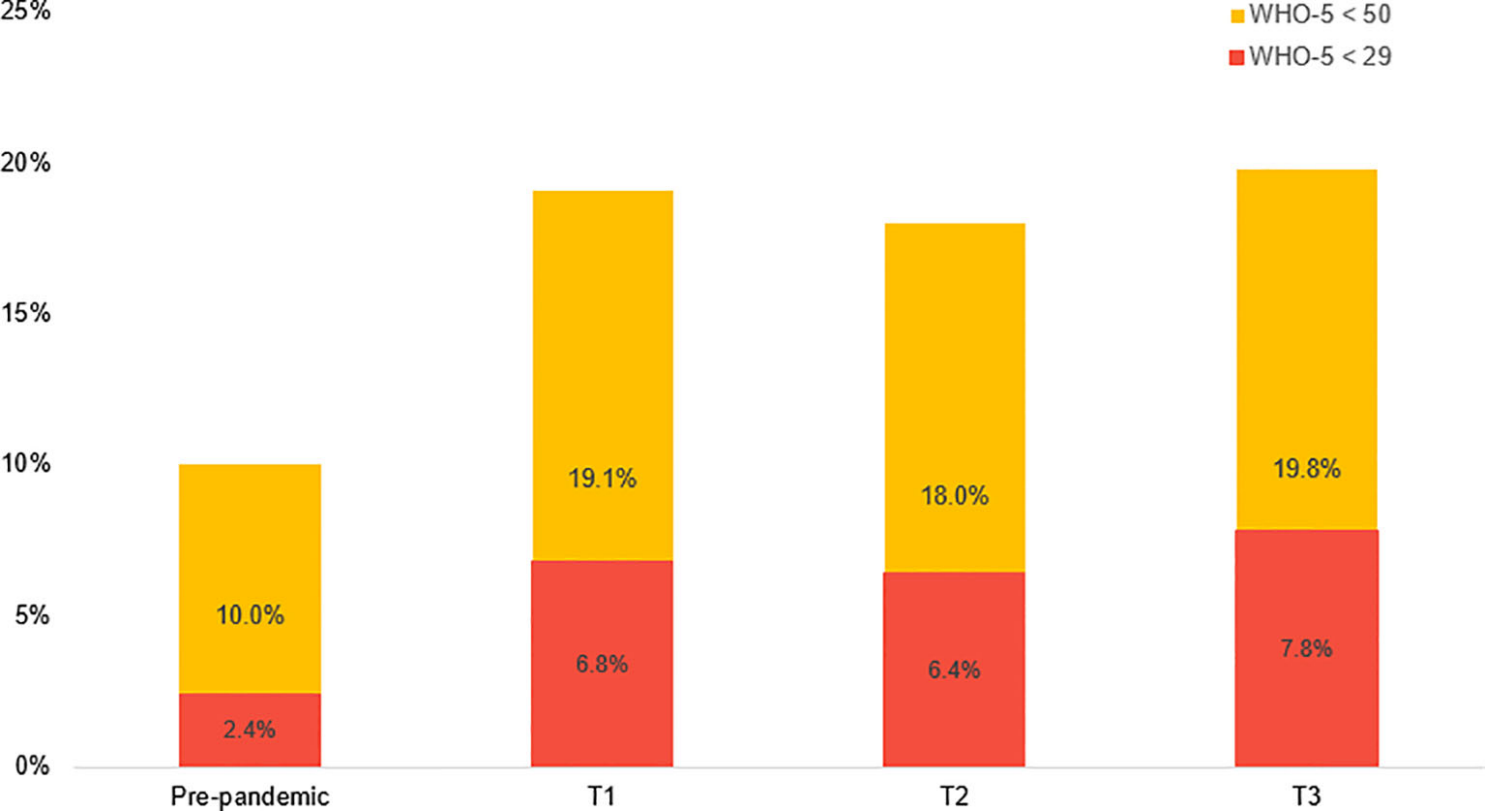
Representation of the percentage of participants with WHO-5 <50 and <29, indicating depressive symptoms within the two weeks prior to the onset of the pandemic and during the three intra-pandemic waves.

Additionally, subgroup analyses were performed on participants from seven Swiss regions classified according to the NUTS system (Classification of Territorial Units for Statistics; in French nomenclature d’unités territoriales statistiques). For a graphical overview of the seven Swiss regions, see **Figure 1**. Since both dependent variables were not normally distributed according to the Shapiro-Wilk test, and the Levene’s test for homogeneity of variances was significant, a Kruskal-Wallis test for independent samples was used instead of an ANOVA to compare pre- and intra-pandemic well-being and mental health among the seven regions. Bonferroni-corrected *post hoc* analyses were then calculated to assess potential differences in WHO-5 and P-scores across the seven regions.

## Results

**Table 1** presents demographic statistics for all participants from Switzerland across the three combined survey waves (with missing values not included). A total of 5,433 individuals initiated the survey; however, this number was reduced to 4,676 after excluding 757 participants who completed only a few items before discontinuing. The numbers for different variables may vary due to the presence of missing values.

**Table 1:** Demographic data of all participants from Switzerland who took part in the COH-FIT survey between April 2020 and June 2021.

Variable	Value
Age (*m ±* SD, *n*)	45.6 ± 16.5 (4676)
Gender (*n*, %)	2895 (61.9%) Female, 1760 (37.6%) Male, 17 (0.004%) Non-binary, 3 (0.0006%) Transgender or intersex
Country of origin (*n*, %)	2923 (76.3%) Born in Switzerland, 906 (23.7%) Born in other country
Ethnicity (*n*, %)	4427 (94.1%) White 109 (2.0%) Mixed 50 (0.9%) Hispanic 50 (0.9%) Asian 23 (0.4%) African 46 (0.8%) Other
Amount of co-habitants (*m ±* SD, *n*)	2.0 ± 1.5 (4706)
Marital status (*n*, %)	2530 (53.7%) Married or co-living, 1537 (32.6%) Single, 537 (11.4%) Divorced or separated, 108 (2.3%) Widowed
City size (*n*, %)	2077 (44.0%) Village/rural 1565 (33.2%) Small city (10,000–100,000 population) 895 (19.0%) Medium city (100,000–500,000 population) 183 (3.9%) Large city (over 500,000 population)
Employment status (*n*, %)	3289 (70.1%) Paid job 1403 (29.9%) No paid job, including students and pensioners
Socio-economic status (0–100) (*m ±* SD, *n*)	59.9 ± 17.4 (4390)
Education degree (*n*, %)	29 (0.006%) None 190 (4.0%) Primary school 1877 (40.0%) High school 2180 (46.4%) College degree 418 (8.9%) PhD
Healthcare worker (%)	2185 (66.6%) No 1095 (33.4%) Yes
Medical illness diagnosis (lifetime) (*n*, %)	2394 (54.2%) No 2021 (45.8%) Yes
Mental illness diagnosis (lifetime) (*n*, %)	3712 (84.6%) No 677 (15.4%) Yes

The average age of participants in the sample was 45.6 years, with 61.9% identifying as female. 76.3% of participants were born in Switzerland. 77.2% resided in villages, rural areas, or small cities, and more than half were married or cohabiting. The average socioeconomic status was 59.9 ± 17.4 on a scale from 0 to 100, and the level of education was high, with over half of the participants holding a college degree or higher. Regarding health status, 45.8% of participants reported having received a medical diagnosis at some point in their lives, whereas only 15.4% acknowledged having been diagnosed with a mental illness.

The following section provides a detailed analysis of the WHO-5 well-being scores of 4,037 participants across three time waves of the COVID-19 pandemic. In **Table 2**, the well-being scores of the participants, with the sample size indicated in parentheses, are presented as mean values across the three recruitment waves T1, T2, and T3. The lowest WHO-5 values occurred in the first half of 2021. With a score of 75.3, the pre-pandemic well-being score was higher than the scores recorded during the pandemic. In addition to the average WHO-5 score of the participants during each survey wave, the percentage change compared to the pre-pandemic score is also provided in parentheses. The average WHO-5 score across the whole sample decreased to 66.5 (−11.7%) during the first wave, measured 69.1 (−8.2%) during the second wave, and 65.1 (−13.5%) during the third wave.

**Table 2:** Average intra- and pre-pandemic WHO-5 during the three survey waves T1, T2, and T3.

Pre-pandemic (February–March 2020) *m ± SD (n)*	T1 (April–June 2020) *m ± SD (n)*	T2 (July–December 2020) *m ± SD (n)*	T3 (January–June 2021) *m ± SD (n)*
75.3 ± 18.5 (4037)	66.5 ± 23.3 (1366)	69.1 ± 22.9 (1286)	65.1 ± 24.0 (1216)

**Figure 1** visualizes WHO-5 with the two bars representing the percentage of participants who scored <50 and <29 in each of the intra-pandemic waves, as well as the cumulative data for all participants in the two weeks prior to the onset of the pandemic. The highest percentage of individuals at risk for depression occurred during the third wave in 2021, with 19.8% of participants reporting WHO-5 <50, followed closely by the first time period from April to June 2020, where 19.1% reported the same. Between 6.4% and 7.8% of participants reported intra-pandemic WHO-5 below the cutoff of 29, indicating a risk of developing major depression. In contrast, before the pandemic, only 10.0% of individuals reported WHO-5 <50, and just 2.4% reported scores <29.

The following section examines the mental health burden, as measured by the P-score, across the three waves, as summarized in **Table 3**. Elevated scores indicate an increase in mental health issues. The pre-pandemic P-score was calculated for all participants.

**Table 3:** Average intra- and pre-pandemic P-scores during the three survey waves T1, T2, and T3.

Pre-pandemic (February–March 2020) *m ± SD (n)*	T1 (April–June 2020) *m ± SD (n)*	T2 (July–December 2020) *m ± SD (n)*	T3 (January–June 2021) *m ± SD (n)*
20.6 ± 17.5 (3375)	27.3 ± 20.0 (1114)	26.0 ± 20.6 (1171)	26.5 ± 20.3 (1037)

Our findings demonstrate that participants experienced the most significant decline in mental health during the first wave, with an average P-score increase from 20.6 to 27.3 (+32.5%) compared to pre-pandemic outcomes. This was followed by an increase to 26.5 (+26.2%) during the second wave and 26.0 (+28.6%) during the third wave, demonstrating that the elevated P-score values did not recover during those waves.

WHO-5 and P-scores from the first (*n* = 1345) and second (*n* = 1282) waves were compared to those recorded before the pandemic via Wilcoxon test for paired samples.

The Wilcoxon test showed a statistically significant difference between pre-pandemic and intra-pandemic WHO-5 reported by participants during the first wave in spring 2020, with *Z* = 15.809 (*p* < 0.001, *n* = 1345) and an effect size of *r* = 0.431, indicating a moderate to large effect. Similarly, P-scores before and during COVID-19 differed significantly (*Z* = −12.937, *p* < 0.001, *r* = −0.398, *n* = 1055), reflecting a moderate effect.

In the second wave, although participants reported improvements in well-being and mental health, intra-pandemic WHO-5 remained lower in comparison to pre-pandemic levels (*Z* = 11.383, *n* = 1282, *p* < 0.001; *r* = 0.318), suggesting a small to moderate effect. Additionally, a significant difference was detected for the P-score (Z = −8.967, *n* = 1150, *p* < 0.001; *r* = −0.264), suggesting a small effect.

During the third wave, the difference between pre-pandemic and intra-pandemic WHO-5 showed a large increase (*Z* = 22.586, *p* < 0.001, *n* = 1195, *r* = 0.652). Similarly, P-scores also demonstrated a large difference in favor of the pre-pandemic value (*Z* = −15.290, *p* < 0.001, *n* = 990, *r* = 0.487).

The results of the ANOVA revealed a significant interaction between the intra-pandemic time period and WHO-5 (F(2, 3865) = 9.638, *p* < 0.001), with a partial eta squared (*η*² = 0.005) indicating a small effect size. Notable differences in wellbeing were observed across the time periods according to the Sidak *post hoc* test: Participants reported significantly higher WHO-5 during the second wave compared to the first wave (*p* = 0.012), with an average increase of 2.6 points on the WHO-5 scale. Furthermore, WHO-5 during the second wave were also significantly greater than those during the third wave (*p* < 0.001), showing an average difference of 4.0 points. Importantly, no significant difference in wellbeing was noted between the first and third wave (*p* = 0.334).

In contrast, the second ANOVA did not reveal any significant differences in mental health burden (P-score) across the three intra-pandemic waves (F(2, 3319) = 1.212, *p* = 0.298, *η*² = 0.001).

Additionally, a Kruskal-Wallis test confirmed the ANOVA results, indicating significant differences between intra-pandemic time periods for wellbeing (*p* < 0.001), but not for the P-scores (*p* = 0.104).

The upcoming section explores regional analyses of well-being and mental health impacts during the COVID-19 pandemic, starting with an overview of the seven Swiss regions as outlined in **Table 4**. This table provides descriptive details about each region, including region codes, names, cantons, official languages, population, and the number of survey participants.

**Table 4:** Descriptive information about the seven Swiss regions and their proportion of survey participants.

Region code	Region name	Cantons	Official languages	Inhabitants (*n, %*)	Survey participants (*n, %*)
CH01	Lake Geneva region	Geneva, Wallis, Waadt	French, German	1,726,237 (19.3%)	1,443 (27.3%)
CH02	Espace Mittelland	Berne, Fribourg, Jura, Neuchâtel, Solothurn	French, German	1,940,275 (21.7%)	1,326 (25.1%)
CH03	Northwestern Switzerland	Aargau, Basel-Landschaft, Basel-Stadt	German	1,220,869 (13.7%)	548 (10.4%)
CH04	Zurich	Zürich	German	1,601,446 (17.9%)	704 (13.3%)
CH05	Eastern Switzerland	Appenzell Ausserrhoden, Appenzell Innerrhoden, Glarus, Grisons, Schaffhausen, St. Gallen, Thurgau	German, Romansh	1,233,817 (13.8%)	580 (11.0%)
CH06	Central Switzerland	Lucerne, Nidwalden, Obwalden, Schwyz, Uri, Zug	German	851,373 (9.5%)	281 (5.3%)
CH07	Ticino	Ticino	Italian	357,289 (4.0%)	404 (7.6%)

Source: Permanent resident population by nationality category, age, and canton at the end of the third quarter of 2023. BFS number cc-d-01.02.03.02. STATPOP – Quartalsproduktion, Bundesamt für Statistik (BFS), Demography and Migration Section (https://www.bfs.admin.ch/bfs/de/home/statistiken/katalog.assetdetail.33248190.html). Status as of November 9, 2023.

The survey sample is mostly representative, with participant numbers corresponding to the population share of the region in the total population of Switzerland. The map of Switzerland with its seven major regions can be found in the **Supplementary Material** for more details.

**Table 5** displays the WHO-5 mean across the three recruitment waves, T1, T2, and T3, in the seven Swiss regions with the number of participants given in parentheses. The results from the entire sample are shown above in bold for better comparability.

**Table 5:** Average intra- and pre-pandemic WHO-5 during the three survey waves T1, T2, and T3 in the whole sample and in seven Swiss regions.

Swiss region code	Pre-pandemic (February–March 2020) *m ± SD (n)*	T1 (April–June 2020) *m ± SD (n)*	T2 (July–December 2020) *m ± SD (n)*	T3 (January–June 2021) *m ± SD (n)*
Total sample	75.3 ± 18.5 (4037)	66.5 ± 23.3 (1366)	69.1 ± 22.9 (1286)	65.1 ± 24.0 (1216)
CH01	74.5 ± 19.3 (1087)	65.4 ± 24.0 (532)	66.6 *±* 24.0 (282)	64.4 ± 24.0 (242)
CH02	76.3 ± 18.3 (979)	68.4 ± 23.5 (415)	70.8 *±* 22.9 (281)	65.9 ± 24.2 (252)
CH03	74.9 ± 17.2 (432)	66.1 ± 20.9 (107)	68.4 ± 23.2 (177)	61.4 ± 25.8 (130)
CH04	74.8 ± 17.6 (537)	64.2 ± 22.4 (148)	69.8 ± 22.5 (233)	65.7 ± 20.6 (140)
CH05	76.5 ± 18.2 (432)	68.2 ± 22.3 (85)	72.9 ± 22.0 (175)	64.7 ± 23.4 (112)
CH06	74.8 ± 19.0 (209)	70.6 ± 23.4 (19)	66.4 ± 20.1 (100)	63.9 ± 25.8 (79)
CH07	73.6 ± 19.9 (265)	59.2 ± 27.0 (23)	64.8 ± 26.3 (29)	65.2 ± 24.2 (213)

Ticino (CH07), the Lake Geneva region (CH01), and Northwestern Switzerland (CH03) exhibited the most substantial declines. In Ticino, the average WHO-5 score decreased markedly from 73.6 ± 19.9 to 59.2 ± 27.0 (−19.6%). Similarly, the Lake Geneva region’s scores were consistently lower than the overall average, with values recorded at 66.6 ± 24.0 during the second wave and decreasing to 64.4 ± 24.0 in the third wave. Northwestern Switzerland reported the lowest WHO-5 score during the third wave at 61.4 ± 25.8. In contrast, while the regions of Espace Mittelland (CH02) and Eastern Switzerland (CH05) also experienced declining WHO-5 during the three pandemic waves, they maintained comparatively higher scores throughout the pandemic, indicating better overall well-being relative to other regions and the average of the entire sample.

The percentage of individuals reporting WHO-5 values <50 and <29 was used as an indicator of depression risk (41, 45, 50), and this was also calculated for the seven Swiss regions. The regions with the highest proportions of individuals with well-being scores below cutoff (WHO-5 < 50) included Ticino (CH07), where 20.7% to 52.2% of participants were identified as at risk for depression, with 11.7% to 13.8% scoring below 29 on the WHO-5 scale. In the Lake Geneva region (CH01), over 25% of participants consistently reported WHO-5 <50, indicating depression risk, while in Northwestern Switzerland (CH03), the at-risk population varied between 20.6% and 34.6%.

The course of the P-score during the COVID-19 pandemic in the seven Swiss regions is presented in **Table 6**. Throughout all three time waves, residents of Ticino (CH07) and the Lake Geneva region (CH01) reported numerous mental health problems, as indicated by higher P-scores. The highest mental burden was experienced by participants from Ticino (CH07) between January and June 2021. During this period, the P-score worsened from 22.0 (± 17.5) to 31.5 (± 21.9), representing an increase of 43.2%. In contrast, the group surveyed in the second half of 2020 reported less mental health problems. In comparison to the Swiss average for the entire sample, a lower mental health burden was observed in Espace Mittelland (CH02), Northwestern Switzerland (CH03), and Eastern Switzerland (CH05) across all three time waves. Residents of the Zurich region (CH04) initially reported a high mental health burden during the early phase of the pandemic; however, participants in subsequent phases showed improved mental health, with P-scores nearly returning to pre-pandemic levels. In Central Switzerland (CH06), while participants questioned from April to June 2020 and those in 2021 had comparatively low P-scores, those surveyed in late 2020 reported the highest P-score during that survey period.

**Table 6:** Average intra- and pre-pandemic P-scores during the three survey waves T1, T2, and T3 in the whole sample and in seven Swiss regions.

Swiss region code	Pre-pandemic (February–March 2020) *m ± SD (n)*	T1 (April–June 2020) *m ± SD (n)*	T2 (July–December 2020) *m ± SD (n)*	T3 (January–June 2021) *m ± SD (n)*
Total sample	20.6 ± 17.5 (3375)	27.3 ± 20.0 (1114)	26.0 ± 20.6 (1171)	26.5 ± 20.3 (1037)
CH01	21.4 ± 17.6 (888)	28.9 ± 21.2 (419)	28.9 ± 21.8 (256)	28.7 ± 20.8 (205)
CH02	20.3 ± 17.4 (821)	26.3 ± 18.8 (342)	25.0 ± 20.0 (258)	26.3 ± 21.4 (220)
CH03	20.6 ± 17.5 (352)	25.4 ± 19.9 (95)	24.6 ± 19.8 (155)	26.7 ± 20.4 (98)
CH04	20.9 ± 18.0 (454)	27.7 ± 20.0 (122)	25.4 ± 21.2 (217)	21.2 ± 15.0 (119)
CH05	18.4 ± 17.2 (368)	25.8 ± 20.0 (74)	22.1 ± 19.0 (157)	24.6 ± 19.1 (97)
CH06	21.8 ± 18.2 (184)	25.3 ± 19.3 (17)	30.0 ± 21.0 (93)	23.4 ± 19.1 (69)
CH07	22.0 ± 17.5 (227)	28.6 ± 21.5 (18)	24.5 ± 18.6 (26)	31.5 ± 21.9 (187)

The results indicate significant declines in well-being and an increase in mental health burden across the seven regions, with Ticino (CH07), the Lake Geneva region (CH01), and Northwestern Switzerland displaying the most pronounced changes. In Ticino (CH07) specifically, the WHO-5 score decreased from the pre-pandemic 73.6 ± 19.9 to 59.2 ± 27.0 during the first wave, reflecting a decline of 19.6%. Concurrently, the P-score exhibited a worsening trend, increasing from the pre-pandemic value of 22.0 ± 17.5 to 31.5 ± 21.9 (+43.2%) during the third wave. Both the WHO-5 well-being scores and the P-scores were compared across Swiss regions using the non-parametric Kruskal-Wallis test for independent samples, followed by *post hoc* analyses to assess whether the deterioration in these measures occurred uniformly across regions.

Results indicated regional differences in well-being, as measured by the WHO-5, across the three periods. At T1, no significant differences between regions were observed (H(6) = 10.230, *p* = 0.115). However, pairwise comparisons revealed differences between Espace Mittelland (CH02) and Zurich (CH04), and between Lake Geneva (CH01) and Espace Mittelland (CH02). At T2, significant differences emerged: participants from the Lake Geneva region (CH01) reported lower well-being compared to those from Eastern Switzerland (CH05) and Espace Mittelland (CH02). Additionally, participants from Central Switzerland (CH06) reported lower well-being than those from Eastern Switzerland (CH05) and Espace Mittelland (CH02). By T3, no significant regional differences in WHO-5 scores were detected (H(6) = 4.262, *p* = 0.641).

Regarding the P-scores, no significant regional differences were found at T1 (H(6) = 3.753, *p* = 0.710). However, at T2, significant differences emerged (H(6) = 13.480, *p* = 0.031), with Central Switzerland (CH06) exhibiting higher P-scores than Espace Mittelland (CH02) and Eastern Switzerland (CH05). Additionally, the Lake Geneva region (CH01) reported worse mental health burden than Eastern Switzerland (CH05). At T3, significant regional differences were observed: inhabitants of Ticino (CH07) exhibited higher mental health burden than those from Espace Mittelland (CH02), Zurich (CH04), Eastern Switzerland (CH05), and Central Switzerland (CH06). Furthermore, the Lake Geneva region (CH01) showed higher P-scores than Zurich (CH04) and Eastern Switzerland (CH05).

No significant differences were found for both the pre-pandemic WHO-5 values (H(6) = 12.041, *p* = 0.061) and the pre-pandemic P-score (H(6) = 10.224, *p* = 0.116). The pre-pandemic values were aggregated across all three periods.

## Discussion

In our Swiss convenience sample, participants reported higher well-being prior to COVID-19, with an average WHO-5 score of 75.3, compared to lower scores during the pandemic. Well-being was lowest from January to June 2021 (mean 65.1), following a decline from April to June 2020 (66.5), but showed improvement during July to December 2020 (69.1). The mental health burden, measured by the P-score, remained relatively stable across waves (26.0–27.3), with a better pre-pandemic value (mean 20.6). The international sample analyzed by Solmi, Thompson, et al. (40) reported a lower average WHO-5 of 60.4, reflecting a more pronounced decline from a pre-pandemic average of 71.5, and a higher intra-pandemic P-score of 40.7 contrasting a pre-pandemic with a P-score of 27.5.

Our findings indicate fluctuating well-being levels amid the pandemic, alongside a relatively stable mental health burden. The apparent steadiness of the P-score could be influenced by several factors. One possibility is that the P-score measurement tool may have limited sensitivity to subtle or short-term changes in mental health indicators, thereby masking transient fluctuations that participants experienced. Alternatively, it is conceivable that the core aspects of the mental health burden assessed by the P-score, such as markers of mental illnesses, are inherently stable traits in this population, less prone to rapid change over brief periods. This stability might reflect a resilient baseline mental health status or adaptive coping mechanisms that buffer against acute stressors. Conversely, the intra-pandemic stability of the P-score underscores a significant and persistent deterioration in mental health compared to pre-pandemic assessments, illustrating the impact of COVID-19 on mental health.

Significant differences in WHO-5 and P-scores between pre-pandemic and intra-pandemic periods were observed. The first wave showed moderate to large declines in well-being and mental health, with initial waves leading to heightened stress and anxiety levels (2, 4), while subsequent waves showed improvements (6), though scores still lagged behind pre-pandemic levels. About 19% of participants remained at risk for depression, with 7% suggesting possible major depression throughout the pandemic, suggesting persistent vulnerability in subgroups. Notably, these percentages remained consistent, despite improvements in WHO-5 during the latter half of 2020. On a global scale, the proportion of individuals with WHO-5 scores indicative of depression screening (scores <50) and major depression (scores <29) increased from 13% to 32% and from 3% to 12%, respectively (Solmi, Estradé, et al., 39).

ANOVA results indicated that only 0.5% of the variance in WHO-5 was attributable to measurement timing, highlighting individual differences in well-being trajectories. Future research should explore other factors influencing these fluctuations to better understand mental health dynamics during such crises.

One possible explanation for the fluctuations in well-being could be attributed to the evolving nature of pandemic-related stress. During the first wave, individuals likely faced heightened stress and anxiety due to uncertainty and fear surrounding the virus and abrupt changes in daily life, including restrictions on social and leisure activities. As time progressed, individuals may have adapted to the “new normal” and found ways to cope with the challenges posed by the pandemic, leading to a slight improvement in mental well-being during the second wave from July to December 2020. It is likely that the varying levels of COVID-19 restrictions and measures implemented during the pandemic could explain the observed differences in well-being and mental health burden between time points. For example, the first easing of restrictions in April 2020 may have temporarily alleviated some stress and anxiety for individuals, leading to a slight improvement in well-being during the second wave. The improvement in mental health and well-being reported by participants in our study during the months of July to December 2020 has also been found by researchers in other countries (51–54).

However, as the pandemic worsened in autumn and winter 2020 and continued into 2021, individuals likely faced prolonged stress and isolation, contributing to declines in well-being and increased mental health symptoms. Additionally, other studies (7, 11, 42, 55, 56) emphasize the existence and impact of risk and protective factors, which have led to varying degrees of susceptibility to COVID-19 among individuals.

Overall, the unpredictable nature of the pandemic and the effects of evolving restrictions on people’s daily lives likely contributed to the variations in well-being observed at different times. Our study revealed that while the prevalence of mental health issues, as indicated by the P-score, increased at the beginning of the pandemic and then remained stable throughout the three waves, overall well-being showed fluctuations. This suggests that individuals’ well-being changed in response to varying circumstances.

In Switzerland, the pandemic’s impact on well-being and mental health varied across the seven regions, with regional differences particularly evident during the second wave in late 2020. Participants from Ticino and the Lake Geneva region reported the most severe negative effects (57, 58), while Eastern Switzerland and Espace Mittelland experienced the least decline compared to pre-pandemic status. Notably, all regions indicated better well-being before the pandemic in retrospective assessments. Prior studies found similar pre-pandemic rates of poor self-assessed health and loneliness across German, French, and Italian-speaking areas (7, 59). This consistency suggests that the marked interregional disparities in well-being and mental health emerged specifically during the COVID-19 pandemic.

One possible explanation for the varying impact of the pandemic on mental health among the different regions in Switzerland could be related to differences in the severity and duration of lockdown measures. Additionally, differences in access to mental health services and resources between regions could also play a role in the disparities observed.

Another factor to consider is the cultural nuances and social support systems present in each region (35). Cultural attitudes towards mental health, as well as the availability of social support networks, may have influenced how individuals coped with the challenges brought on by the pandemic.

Overall, the complex interplay of various factors – including lockdown measures, access to resources, cultural norms, social support, and individual differences – has likely contributed to the differing impacts of the COVID-19 pandemic on mental health across Switzerland’s regions. Further research is essential to fully understand the mechanisms underlying these disparities and to develop targeted interventions to support mental well-being in times of crisis.

### Strengths and limitations

The strengths of this study are manifold. Firstly, the extensive time frame allowed for an exploration of changes over time. Moreover, the research covered all regions of Switzerland, providing insights relevant to various geographical contexts. Additionally, it encompasses all national languages spoken in Switzerland, ensuring a comprehensive representation of the country’s diverse linguistic landscape. The large sample size facilitated subgroup analyses, enhancing the depth of the findings. Lastly, the study’s comparability with international samples (39–43) enabled a broader understanding of the evolution of well-being and mental health burdens during the COVID-19 pandemic.

All surveys have limitations, some of which are outlined below. While the study aims to represent the Swiss population, certain demographic and socio-economic factors may restrict the generalizability of the results. Selection effects may also have played a role, given the higher percentage of women, the high level of education in the sample and the low percentage of individuals reporting notably low well-being (defined as WHO-5 < 50). This may be attributable to convenience sampling as well as dissemination through media releases from universities and the media.

For instance, the high level of education observed in the sample may not accurately reflect the broader population, as individuals with lower educational attainment or socio-economic status may have different experiences related to well-being and mental health during the pandemic. This selection effect could skew the findings, making them less applicable to underrepresented groups.

Additionally, while the study included diverse geographical regions, variations in COVID-19 measures and local responses across cantons may introduce discrepancies in participants’ experiences. Comparison of well-being and mental health burden across Swiss regions was challenging due to variations in participant numbers in each area and survey time period. For the detailed analysis of the individual cantons, the already varying case numbers were sometimes too low. The differing levels of strictness in public health measures, along with varying infection rates, could lead to context-specific impacts on mental health that are not fully captured in the overall analysis. Therefore, the results may not be entirely representative of the experiences of all Swiss residents, particularly those in cantons that faced more severe restrictions or different socio-economic challenges.

Furthermore, the reliance on recall for well-being prior to the pandemic introduces the possibility of recall bias, which could affect the accuracy of participants’ responses. Changes in external circumstances, such as infection rates and governmental measures, can also impact well-being and mental health with a time delay or due to anticipatory effects, complicating direct correlations (60–62). This temporal aspect may hinder a clear understanding of causality between these external factors and mental health outcomes.

Despite these challenges, the study offers valuable insights into the effects of the COVID-19 pandemic on mental health and well-being in Switzerland.

## Conclusions

This study highlights the COVID-19 pandemic’s significant negative impact on mental well-being in Switzerland, particularly during the early months and the second wave in early 2021. A marked deterioration in intra-pandemic WHO-5 outcomes and P-scores and a 19% risk of depression among participants highlights urgent mental health challenges. Well-being fluctuated with pandemic severity and restrictions, likely reflecting varying individual coping strategies. Regional disparities in mental health outcomes indicate the influence of lockdown intensity, cultural perceptions, and resource access. Although some recovery occurred in late 2020, lasting psychological effects remain a concern. Future research should explore long-term trends in well-being, effects of restrictive measures by the government and other institutions on well-being and mental health, demographic responses to recovery, and individual coping strategies. Follow-up studies are crucial, especially in heavily affected regions like Ticino and Lake Geneva, to assess current mental health compared to less impacted areas. Evaluating the effectiveness of mental health support strategies and fostering collaboration among stakeholders will be vital in developing comprehensive frameworks for mental health support in future crises, ultimately strengthening societal resilience against unprecedented challenges.

## Data Availability

The raw data supporting the conclusions of this article will be made available by the authors, without undue reservation.

## Supplementary material

Supplementary Figure 1 Excess mortality: Cumulative deaths in Switzerland from all causes compared to projection based on previous years from March 1, 2020 until July 4, 2021. Data source (26): (https://github.com/akarlinsky/world_mortality; https://github.com/dkobak/excess-mortality).

Supplementary Figure 2 Cumulative confirmed COVID-19 cases and deaths in Switzerland from March 1, 2020 until June 30, 2021. Data source (63): (https://covid19.who.int) – processed by Our World in Data.

Supplementary Figure 3 Oxford COVID-19 stringency index of COVID-19 measures in Switzerland from February 29, 2020 until June 30, 2021. Data source: https://ourworldindata.org/explorers/coronavirus-data-explorer (33).

Supplementary Figure 4 Seven Swiss regions and cantons (Grossregionen und Kantone der Schweiz). Source: Bundesamt für Statistik (https://www.bfs.admin.ch/bfs/de/home/statistiken/querschnittsthemen/raeumliche-analysen/raeumliche-gliederungen/analyseregionen.assetdetail.1031445.html).
