# Supplementary figures and images for: Course of well-being and mental health in Switzerland during the COVID-19 pandemic: results of a national survey within the framework of the COH-FIT study
Source: Front Psychiatry. 2026 Feb 16;16:1642325. doi: 10.3389/fpsyt.2025.1642325 (PMC12950789; doi:10.3389/fpsyt.2025.1642325)

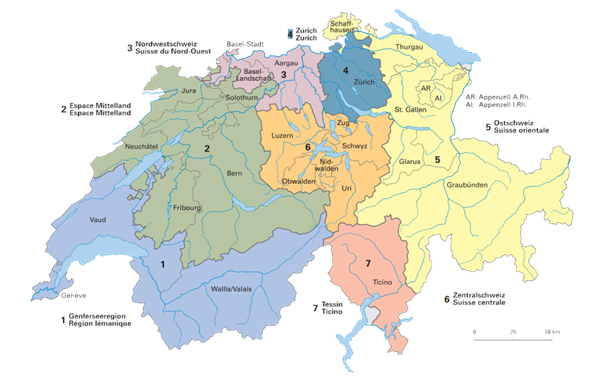

Supplement: Supplementary Figure 1 — Excess mortality: Cumulative deaths in Switzerland from all causes compared to projection based on previous years from March 1, 2020 until July 4, 2021. Data source (26): (https://github.com/akarlinsky/world_mortality; https://github.com/dkobak/excess-mortality). [file Image1.png]

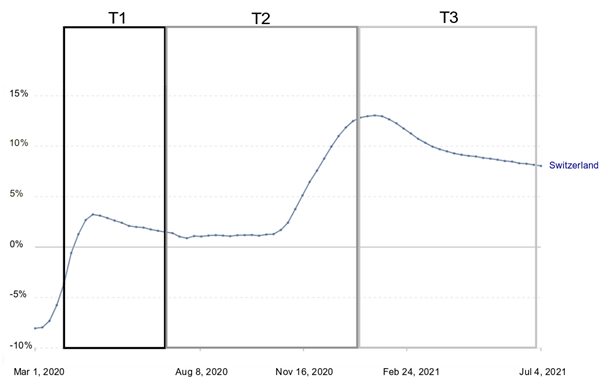

Supplement: Supplementary Figure 2 — Cumulative confirmed COVID-19 cases and deaths in Switzerland from March 1, 2020 until June 30, 2021. Data source (63): (https://covid19.who.int) – processed by Our World in Data. [file Image2.png]

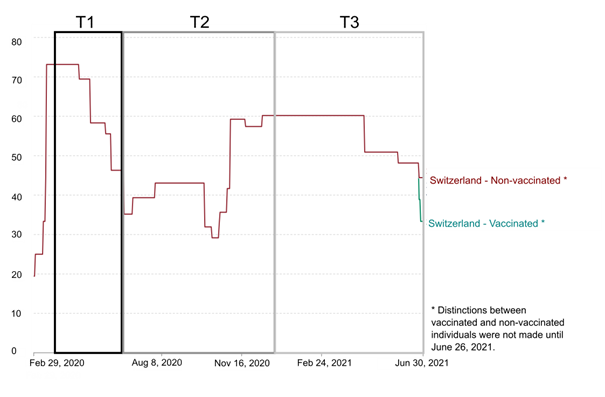

Supplement: Supplementary Figure 3 — Oxford COVID-19 stringency index of COVID-19 measures in Switzerland from February 29, 2020 until June 30, 2021. Data source: https://ourworldindata.org/explorers/coronavirus-data-explorer (33). [file Image3.png]

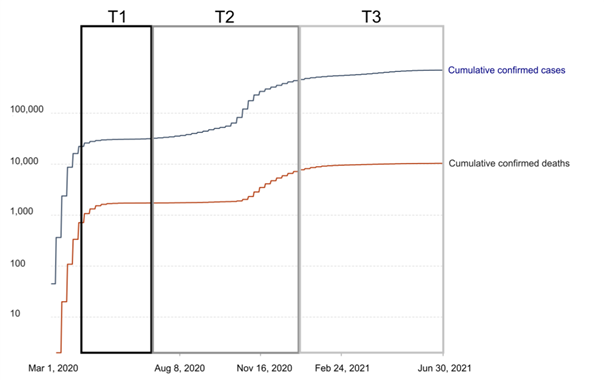

Supplement: Supplementary Figure 4 — Seven Swiss regions and cantons (Grossregionen und Kantone der Schweiz). Source: Bundesamt für Statistik (https://www.bfs.admin.ch/bfs/de/home/statistiken/querschnittsthemen/raeumliche-analysen/raeumliche-gliederungen/analyseregionen.assetdetail.1031445.html). [file Image4.png]
